# On the Intellectual and Moral Character of the Age

**Published:** 1853-04-01

**Authors:** 


### Art. IV.—ON THE INTELLECTUAL AND MORAL CHARACTER OF THE AGE.

The intellectual and moral development of the present age. What a scene does this open! What a magnificent theme for a Herschel or a Chalmers, and yet how dangerous a one to any lesser intelligence! How seductive to an unbridled imagination! How enticing to an inferior mind fond of melodramatic effect, delighting in turgid exclamations, mistaking sound for sense, and revelling in elaborate and involved sentences. What more appropriate text could be chosen by any one who mistakes the pomp and parade of mere words for eloquence, or grandiloquent declamation for oratory! It is a subject sufficiently difficult for any mind, however great its attainments, or profound its judgment; and yet sufficiently easy to suit the purpose of the flimsiest pedant. Like the science of astronomy, it may serve for the life-study of a Newton, or supply a gaudy oration for the vapid declaimer of a debating club. To whether of these categories does the book before us belong?* Is it the analysis of a profound thinker, or the plausible oration of a declaimer? Do we perceive in it the calm deductions of a philosopher, or the special pleadings of the advocate? The pompous phrases of the rhetorician, or the gathered facts and clear inductive reasonings of a scientific man? We will deal faithfully with this question. The book is small in size, but great in its pretensions. The circumstances under which it was produced, the place in which its contents were publicly read, the reputation, and dignified position of the author, and above all the importance of the subject, claimed and

* "The Intellectual and Moral Development of the Present Age." By Samuel Warren, F.R.S., one of Her Majesty's Counsel, and Recorder of Hull. Blackwood.

gained from us the most respectful attention. We opened its pages with reverence, and expected much instruction. We have learnt that the author in his first judicial visit to Hull as its Recorder, received an unexpected and earnest request from the President and Council of the Literary and Philosophical Society of that place to read a paper before the society, upon any subject which he might select. He writes—"After much consideration, I expressed my willingness to do so, and chose the subject now before us. Some time afterwards I was honoured by receiving a unanimous resolution of the President and Council, soliciting me to take steps, by anticipation, to commit the paper to the press, in order that it may be perused at as early a period as possible, by those who cannot hear the paper read, with a view to its extended usefulness."—(page 2.) We, however, were more especially interested in the work, because profound metaphysicians, "subtle, learned, and original thinkers," had been consulted as to the present condition of mental philosophy; and distinguished inquirers into physical science had given up, "for the purposes of the paper," some valuable facts and inductions, which were otherwise intended "for private circulation only."

Moreover, the author assures us, that he uses the word, development, in its strict etymological signification; that is to say, "an opening," a "showing forth," "a displaying" of the intellectual and moral condition of man in the present age. And you will say, is this to be done in a single evening's paper? It sounds, indeed, as hopeless as the notion of compressing the Iliad into a nut-shell. Nevertheless, the attempt must be made to survey this vast field, however rapidly, and however hard it may be to know where to begin. The great object is for the observer to select *a right point of view.* On that depends everything; for there *is* a point from which everything within and without is order and loveliness; and another from which all is contradiction and confusion. There is a string which, *untuned,* we may well call out fearfully,

> "Hark, what discord follows!"—p. 9.

Prior to the selection of this point of view, the author had informed us, that the paper contained some "of the results of nearly a quarter of a century's observation and reflection," and that

"We cannot survey, for the purpose of practically estimating, *the intellectual and moral development of the age* in which we live and are playing our parts,—every man and woman of us having his or her own responsible mission to perform,—without attempting gravely and comprehensively to consider man in ordained relation to his power and his knowledge, his objects, his sayings and his doings, his position past, and present, and his destiny. . . . . It will be necessary, with this view, to soar high and far, but swiftly, into the stupendous starry solitude of space; to descend, as far as man's limited means allow him, into the interior of the earth; and again, to travel all round its surface, in order to ascertain what we know, or think we know, of the human and animal denizens of that earth itself; and, finally, to penetrate, as far as we may, and with tender respect, into that mystery of mysteries, MAN himself."

The paper achieving this, "somewhat modified and amplified, is now presented to the public." There is no amount of verbal pomp too great to usher in such a magnificent result. The highest ornaments of composition, the boldest apostrophes, the most sublime personifications, are not misplaced in the opening of *such* an address. The severest criticism, nay, the very shade of Swift himself, would recognise, for instance, the chaste propriety of the following exordium at page 3—

> "Be still, ye winds! ye zephyrs, cease to blow,
> While music most melodious meets my ear, the 'still sad music of humanity,' which may be heard echoing while we fix our eyes upon MAN and his mysterious manifestations—in his momentous relations to the Past, the Present, and the Future."

But alas! despite these loud pretensions, this invocation to the winds and the zephyrs, this determination to climb the starry heights, to plunge deep into the abysses of the earth, and further, like Ariel himself, to girdle the round world, to "scan its human and animal denizens," and to penetrate "into that mystery of mysteries, Man himself," we gain very little by the marvellous tour. To all who have read (and who has not?) the masterly discourse of Herschel on the study of Natural Philosophy, the recent treatise of the industrious Astronomer at the Regent's Park Observatory, and Mr. Owen's productions, the work will prove neither novel nor profound. It is meagre of new facts, and offers no explanation to the vast intellectual and moral development of the present age. Notwithstanding its pretentious assumption of the philosophic spirit, it does not attempt to generalize the borrowed facts which it has derived from the pages above referred to. It stands in painful contrast to the beautiful essay of Dr. Channing "On the Present Age;" for although it glitters with a formidable array of scientific facts derived from the sources above indicated, yet, unlike the essay of Dr. Channing, it is destitute of any general principle, and fails to grasp the chief characteristics of the *present* age, as distinguished from the ages which have preceded it. Whenever the writer departs for a moment from the works and writings of others, that moment he falls into error; and this is nowhere more conspicuous than when, with an affectation of philosophic caution, he discourses on the progress of mental philosophy, and apostrophises

"The Intellect," with all the energy and force which the printer can supply to him by means of exclamatory marks and notes of interrogation. But we are forestalling our observations upon this subject. The transcendant abilities of this author as a writer of fiction, entitle him to be heard most fully, and with respectful attention; and although a careful perusal of the essay has filled us with intense disappointment, yet gratitude for his past achievements prompts us to place statements before our readers which, falling from a man of less renown, would pass unheeded. The author first treats of English composition:

"I say these things only for the advantage of the younger portions of this large audience, and of those who may hereafter think it worth while to read what I am now uttering; and to them would that I could speak trumpet-tongued on this subject, which has always lain near my heart. Let them believe the assertion, which will be readily supported by the greatest masters of our language, that to write English with vigour and purity is really a high, and also a rare accomplishment: much rarer, indeed, than it ought to be, and would be, if youthful aspirants would only conceive rightly, and bear ever in mind the importance of the object, and the efforts indispensable to secure it. This accomplishment involves, in my opinion, early and careful culture, continued attention, and sedulous practice, familiarity with the choicest models, and no inconsiderable degree of taste and refinement. One thus endowed and accomplished must sometimes shudder at the extent to which he may see our language vitiated by needless and injurious incorporation of foreign words and idioms, and vulgar fleeting colloquialities of our own viler growth, which are utterly inconsistent with the dignity of high and enduring literature. Any man of talent, or more especially of *genius* (a distinction difficult to put into words, but real and great, and not in degree, but kind), who disregards these considerations, offends the genius of English letters; and, indeed, let him rest assured, commits a sort of literary suicide. He may be unconsciously disgusting thousands—nay, tens of thousands, of persons competent to detect at an indignant glance these impertinent and vulgar departures from propriety: familiar with the finest models of ancient and modern literature; persons, in short, whose estimation constitutes the true and only pathway to posterity. If their *fiat*, or *imprimatur*, be withheld (and it is given only after a stern scrutiny), the eager, ambitious traveller will by and by find out, to his mortification, that he has started *without his passport*."—pp. 15, 16.

The italics belong to the author.

We recognise the truth and importance of the idea which is sought to be enforced by the above extract. Moreover, the passage itself is a proof that "to write English with *vigour* and *purity*" is "a rare accomplishment;" and "we shudder at the extent" to which its purity has been "vitiated by needless and injurious incorporations" of such "foreign words and idioms" as "colloquialities," "fiat," "imprimatur,"

and the like. And we further think, that its "vigour" has been diminished by a too redundant use of adjectives—the struggle to be forcible is too conspicuous, especially towards its close; matter is wanting, and sound is substituted; and the energy, such as it is, resembles the convulsions of disease or debility, rather than the serene power of a healthy man.

The author expresses a fear that the number of writers in the present day bear too great a proportion to the readers; but thinks that it cannot be denied, that the current of our periodical literature is highly creditable to an accomplished age. We mourn over the truth of the following statements:

"It is a fact, however, stated with concern and reluctance, that there is a poisonous growth of libertine literature—if the last word be not indeed libelled by such a use of it—designed for the lowest class of society; supplied, moreover, to an extent scarcely equal to the demand for it, and which exists to an extent unfortunately little suspected. I know not how this dreadful evil is to be encountered, except by affording every possible encouragement, from every quarter, to the dissemination in the cheapest practical form of wholesome and engaging literature. If poison be cheap, let its antidote be cheaper."—p. 19.

He might have added, that vigorous efforts have been made to supply the antidote; and that great as are the exertions of the infidel, they are equalled by the zeal and industry of the various religious societies of "the present age." The British and Foreign Bible Society is issuing its productions by hundreds of thousands; and many minor societies are actively engaged in the same holy undertaking. Indeed, while we mourn the truth of the author's statements, we are cheered by the conviction, that the evil to which he refers is diminishing, and that socialism and its twin sister infidelity are not so vigorous as they were a few years ago. Dr. Vaughan, in his "Age of Great Cities," quotes the following:

"I think, and indeed I have very good reason to believe, that the progress of socialism and infidelity has been effectually checked in the manufacturing districts. . . . . In the houses of the needy and the afflicted, I have often found the Bible the last piece of furniture remaining. I have heard the miserable proclaim the patience they have learned from its precepts, and the consolation they have derived from its promises. If any man doubted the benefits which Christianity has conferred on mankind, he could be cured of his scepticism by witnessing its soothing influence on the distress and suffering in Lancashire."

However, evil is an active agency; and it behoves all those who wish well to their fatherland, and to the highest interests of their fellowmen, to do what in them lies to stem the torrent of unbelief, for with it flows the germs of anarchy, degradation, and national ruin. And this reminds us to observe, that the author has failed to make any remark upon those Mechanics' Institutions which, springing up under the genius of Dr. Birkbeck and Lord Brougham, have now penetrated into nearly every city and town of the United Kingdom. This is the more remarkable, inasmuch as a deputation from the Hull Institute afforded the author peculiar gratification by forming part of his audience, "in pursuance of a liberal and friendly invitation from the President and Council of the Literary and Philosophical Society." We believe that valuable as have been the results of such institutions, they have not been productive of unmixed good, and that to their operations may be fairly ascribed some portion of the heterodox notions to which the author refers. We hope not to be misunderstood, as being opposed to the diffusion of knowledge among the operative classes. Far from it be our every thought and desire. They are, however, human institutions, and therefore cannot be faultless. In a friendly spirit, we desire to point out some evils which we opine have sprung up under their influence. We think, then, that the Mechanics' Institute has tended unduly to exalt mere intellectual attainments, and to elevate secular knowledge above that higher faculty of our nature, which enables us "to eschew evil and to do good." It has, in many minds, kept back that great and cardinal truth, that "the fear of the Lord is the beginning of wisdom," and that "a right understanding have all they that do hereafter." Fascinated by the splendours of poetry, or by the useful results which spring out of well-contrived mechanical adaptations, the ill-educated mind has listened to the conclusion, that a magnificent poem, a fine song, or a skilful invention, were ample compensations for a life of debauchery and sin.

We yield to none in our reverence for great names; and, least of all, would we, with unhallowed hands, tear open the receptacles of the dead to expose the failings and follies of those who moulder there; yet the interests of truth compel us to state, that we have heard the names of Burns, Byron, and others, pronounced with almost idolatrous homage; and to pause for a single moment ere you joined in their unqualified praise, was to call down upon your head the terms of "narrow-minded bigot" and "stupid loon;" however gently you might hint that their moral career was such as to dim their otherwise resplendent characters. This incident is related not as in itself a fearful fact, but as indicating by an example, the tendencies before referred to.

Moreover, the miscellaneous lectures popularly given, involving no labour upon the hearers, produce in them a vain desire to be considered wiser than their neighbours, and to dogmatize upon subjects far beyond the comprehension of the wisest of men. An undue craving for knowledge was the crime which lost the world. Puny scoffers have sneeringly asked, whether it was at all probable that Adam should have forfeited Paradise and "brought death and woe upon all mankind" for the sake of an apple, or any other fruit? To such petty garblings of Holy Writ have men condescended to listen, overlooking the terrible temptation, "to be as gods." To possess mental power, to be endowed with genius, to soar above our fellows in all that pertains to the intellect, was, and is, the strongest temptation which can beset a lofty mind; and the inspired Record therefore harmonizes with every day's experience of the human heart in describing. "Adam, the goodliest man of men since born his sons," falling before the strongest of all temptations, the acquisition of knowledge (power) and the indulgence of his affections. Let us not, however, be understood by the above remarks to advocate the slightest restriction upon the acquisition of any knowledge which it is permitted man to know. We do not, as many, dread the advance of knowledge as conducive to infidelity; far from it; the Bible is based upon truth, and, as it has been often observed, the *words* and the *works* of the Almighty cannot contradict each other. Flimsy and superficial acquirements may beget a giddy self-elevation, and engender a vain conceit which lead on to scepticism; and partial knowledge acquired at second-hand, and from compiled works rather than from the monographs of original thinkers, tends frequently to the same result. On the contrary, profound research and deep thought have ever tended to fill the mind with holy reverence, and to imbue it with an abiding sense of an all-wise and mighty Being, whose wisdom, power, and love pervade and uphold all things. Thus, the poet Shelley, the vulgar sophist Thomas Paine, and the witty Voltaire, could sneer at Revelation as being inconsistent with the sublime dogmas of astronomy, whilst Newton, who had investigated the movements of the celestial bodies, who had, as it were, walked amongst the stars, measured their magnitudes, predicted their movements, and discovered the wondrous law of gravitation, could proclaim with trumpet-tongue as the inspired psalmist had done before him, "The heavens declare the glory of God, and the firmament showeth His handywork." It is so in all science—the smatterers are vain, but the philosophers are reverential and lowly. To revert briefly to our first observations upon this subject, we know not why intellectual power should claim the idolatrous homage of mankind. It has again and again failed in its purpose, when the indomitable *will* has triumphed. Its results have been puny when compared with those which have been effected by a high moral purpose. It was a moral power which thrilled the hearts of our sailors when they nerved their energies to the deathless sentence, "England expects every man to do his duty." It was moral power which actuated Howard and Clarkson in their successful missions of mercy to the felon and the slave; which enabled Pinel to strike off the fetters of the maniac, and empowers his followers to mitigate the sorrows and heal the diseases of this large class of sufferers; and it was the like agency which triumphed over the learning of Erasmus; defied not only the pope, but the demon himself, and won its triumph in the Protestant Reformation.

It was the same influence which animated the soul of our greatest warrior, and melted the hearts of his countrymen, as they stood around his recent grave; this indeed it is, which fills our homes with peace, upholds our laws, and spreads a glory over the length and breadth of our fatherland. Not only have "Mechanics' Institutions" been wholly overlooked in the essay especially devoted to the intellectual and moral development of the age; but (what is more remarkable in an author who takes great pains to show the orthodoxy of his faith, and almost overloads one portion of his essay with scriptural quotations) no prominent notice is taken of the marvellous spirit of missionary enterprise which characterizes the nineteenth century. It is true, that in quoting some singularly-phrased remarks from Chevalier Bunsen's recent work (Hippolytus), we are reminded that Christianity is performing the glorious mission of civilizing the earth, yet the great literary and scientific advantages, which have sprung out of missionary labours, are nowhere referred to. Whatever opinion may be formed respecting the ultimate result of these labours, and of the propriety of directing such energies over so large a sphere of operations, rather than concentrating them upon *the heathendom at home,* this much is certain, that missionary enterprise has extended the boundaries of knowledge, and aided the moral development of the age. It has in many instances formed written languages—in hundreds, extended and improved the languages already existing—it has discovered countries, it has instituted laws—it has exerted a moralizing influence, even where its especial objects has failed, such as in abolishing the Suttees of India, and mitigating the general abominations of heathendom. It has taught useful arts to the Hottentots, to the Esquimaux, and to the South Sea Islanders—it has abolished the migratory habits of many tribes, and implanted the germs of civilization, by teaching uncivilized nations to cultivate and store up the most valuable of the cereal grains, and be thus enabled to possess "a local habitation, and a name." It has added to the stores of literature directly, by bringing new languages and dialects before the Philologists, and science is indebted to it for a vast amount of its ethnographical knowledge. Balbi, speaking of the collection of materials for the comparison of languages, states,—

"In this field, along with many other very useful labourers, the ministers of Christianity have occupied the first rank. To the zeal of the Moravian, Baptist, and other protestant missionaries, as well as to the members of bible societies of all Christian sects, ethnography owes its acquaintance with so many nations hitherto unknown in India, and other regions of Asia, and various parts of America, and Oceanica, along with the translation in whole or in part of the bible, in more than a hundred different languages."*

At the present time the British and Foreign Bible Society has translated the Bible or portions of it, into *nearly* two hundred languages, its volumes embracing dialects of Western, Northern, Central, and Southern Europe; Asia, Africa, North America, and Polynesia formed not the least of the marvels of "that true wonder of our time, that visible and profoundly suggestive epitome and sum of man's doings since he was placed on this planet, the Great Exhibition of 1851."

We might add, also, that our Embassies have gladly availed themselves of the services of missionaries as interpreters of languages, and that learned societies have expressed their obligations to such men as Ellis, Morrison, Gutzlaff, Carey, and others, for information imparted to them on various occasions. In short it is a mighty agency, calling forth the energies of hundreds of able men, and the charities of tens of thousands more, expending, as it does, nearly two-thirds of a million sterling in its beneficent operations—to use the beautiful and philosophic language of Dr. Channing:—

"Call it pretension, or enthusiasm, or what you will, the fact remains; and it attests the diffusive tendencies of our times. Benevolence now gathers together her armies. Vast associations are spread over whole countries for assailing evils, which it is thought cannot be met by the single-handed. There is hardly a form of evil which has not awakened some antagonist effort. Associated benevolence gives eyes to the blind, and ears to the deaf, and is achieving even greater wonders; that is, it approaches the mind without the avenues of eye or ear, and gives to the hopelessly blind and deaf the invaluable knowledge which these senses afford to others. Benevolence now shuts out no human being, however low, from its regard. It goes to the cell of the criminal with words of hope, and in labouring to mitigate public punishment, to make it the instrument, not of vengeance, but reform. It remembers the slave, pleads his cause with God and man, recognises in him a human brother, respects in him the sacred rights of humanity, and claims for him, not as a boon, but as a right, that freedom without which humanity withers, and God's child is degraded into a tool or a brute. Still more, benevolence now is passing all limits of country and ocean. It would send our best blessing to the ends of the earth. It would make the wilderness of heathenism bloom, and join all nations in the bonds of one holy and loving faith. Thus, if we look at the religious movements of

* "Atlas Ethnographique." Paris, 1826.

the age, we see in them that tendency to diffusion and universality, which I have named as its most striking characteristic."*

We think further, that the present *postal arrangements*, as devised by Rowland Hill, have been highly conducive to the moral development of the present age. By it the domestic and social virtues are kept alive, and a ready interchange of thought and argument can be maintained by scientific men, who are located at great distances from each other.

We are living too near the era of extensive fiscal changes to estimate their effects with accuracy, yet without entering upon the stormy and dangerous sea of modern politics, and least of all assenting to the general justice of the sudden repeal of the corn laws, we are bound to confess, that the improvement in the physical condition of the labouring classes has greatly aided in the diffusion of knowledge, and the advancement of public morality. The condition of our workhouses, the comparatively empty state of our gaols, and the general contentment and cheerfulness which pervade the operative classes of this kingdom, may be fairly ascribed to the abundance of employment, and of food; whether that abundance be ascribed to recent legislation, or to the contemporaneous discovery of gold in Australia, and the extensive emigration which has sprung up in consequence thereof.

These great *facts* are not once referred to, but our limits will not permit us to supply the many defects of the author, in treating upon the intellectual and moral characteristics of the present age; and we must limit ourselves to the facts and opinions contained in the work before us. Having made allusions to the number of elementary works and of "sermons and religious publications," of which "he reads and always did read" largely, the author proceeds to allude to biographical writings of which he thinks "a sort of deluge is precipitated upon us," and asks the following pertinent questions:—"Does an indolent and prurient love of *gossip* vitiate the taste of both readers and writers of biography? encouraging the latter to trifle with the memory of the dead, and the intellect of the living?"

The force of the following remarks must be felt by all readers: "I have heard an eminent person say, when conversing on this subject, (the rash publication of private letters.) 'For my part, I now take care to write no letters that may not be proclaimed on the housetops; and am very cautious whom I take into my confidence.'" Is this unreasonable, or unnatural?

We think this a crying vice of the age; and notwithstanding this effusion of private correspondence, we are more deficient in good bio-

*. Channing on "The Present Age," p. 13.

graphical writings than those of any other character. We want works which shall give us a full and succinct portrait of the individual whose life is attempted to be unfolded; mere extracts from journals, and a heterogeneous mass of unimportant letters, no more indicate the man to the every-day reader, than do the disjointed, broken, and scattered fragments of fossil bones reveal the form and size of the "iguanodon," until brought together, compared, and arranged by the genius of a Buckland or a Mantell. How few biographical works of the present age can be compared with Boswell's Johnson, M'Crie's Life of Knox, or the charming portraits of Donne, Hooker, and Wotton, by the honest, quaint, and truthful Isaak Walton?

The essayist thinks it impertinent on the matter of prose fiction "to criticise contemporaries" by way of either censure or praise, "for certain reasons of his own," and therefore limits his observations to a eulogy of Sir Walter Scott; expressing a doubt whether "sufficient pains have been taken in the present day to construct a fiction on a durable basis; and whether there are many that have sufficient vitality to bloom in the atmosphere of the next succeeding century." (p. 33.) This is cautious criticism; "*many*" is a safe word to use upon such a subject. It is indeed such platitudes as these that constitute the basis of the work before us, and so bitterly disappoint its readers, who expect to gain some information from a book, on whose covers are emblazoned, with all the pomp of capital and gilt, the magic symbols of a Queen's Counsel, and a Fellow of the Royal Society; to say nothing of the fact, that *a philosophical society* had requested that steps might be taken "by anticipation to commit the paper to the press" (page 2).

Surely there are writers who symbolize the immediate age, with greater exactness than Sir Walter Scott? And was it to be expected that in treating of the prose fiction of the age no elucidation should be given, or offered, of the wondrous circulation and thrilling effect of "Uncle Tom's Cabin?" Was not a prose fiction which has roused the feelings of *millions*, from the highest aristocrat to the lowliest peasant in the old and new world, worthy of a passing notice? Would it have been inconsistent with the dignity of the author of "The Diary of a Physician," in an essay upon the prose fiction of the present age, to have analyzed a book which is unparalleled in the number of its readers, and the extent of its circulation, by any work of the imagination that has ever preceded it?

We are informed with all the energy of *italics*, that "poetry is not *dead* in the present busy practical age; but her voice is heard only faintly and fitfully, like the sounds of an Æolian harp in a crowded thoroughfare." Why, there is poetry in the above sentence. Sentiment, pathos, original metaphor, and ideal fiction are there. The sentence that mourns its decadence enshrines the hope of its resurrection. Poetry cannot die. Nay more, it cannot *sleep*, for even in the wild blasts of winter its sublime voice is heard, and night with her sable clouds, or gorgeous canopy of stars, calls up its presence to the hearts of men. Spring may no more spread its sunshine over the earth, nor summer clothe the forest, brake, and dell in the rich garniture of leaf and flower; but so long as love throbs in a human heart, and the soul has its aspirations of hope and joy, its moments of bereavement and sorrow, so long will the voice of poetry *be heard*, not only "fitfully," but continuously, and musical as the air which wraps the earth, and fills the listening ear with an ever-varying, but never-dying song. We think, indeed, that the "present age,"—the age which includes Sir Walter Scott as the representative of prose fiction, has been, and *is*, especially *alive* with poetry. Poetry not *dead* indeed! Her voice heard "only faintly and fitfully!" Does the writer expect poets to spring up as abundantly and regularly as mushrooms in autumn? What were the graphic descriptions and gloomy moralizings of Childe Harold, Manfred, and the Corsair? We say nothing of the lyric strains or passionate songs of Burns. Was there none of "the transcendent and glorious faculty," in the rich and glowing effusions of Shelley and Keats? In the wild tale of Thalaba—the philosophic lines of Coleridge, and the gentle lays of Wordsworth, does she sing "faintly and fitfully?" It needed not the energy and force of italics to assure us, that the "Loves of the Angels" and the charming "Irish Melodies" of Moore yet live; and we rejoice to think, that the *Author* of that great epic, "The Lily and the Bee,"—that marvellous poem of the marvellous year of 1851, still lives to adorn and instruct the present age,—to say nothing of such lesser poets as *Rogers*, *Wilson*, Bulwer, *Montgomery*, Browning, Marston, Leigh Hunt, and Tennyson. The author states that the "poetry of the present age is principally and elegantly conversant with *sentiment*, of which it is often a very delicate and beautiful utterance," page 35. We cannot dissent from so general a statement. No special sentiment is referred to, and the poetry of *all ages* is conversant with sentiment. The beautiful odes of Horace, and the sparkling poems of Anacreon possess this quality. It abounds in the odes and choruses of the Greek plays, and flows in an endless stream through the glowing poetry of the Hebrew Canticles. It shines in the pages of Theocritus, and spreads its fascination over the Iliad. Where is more delicate "sentiment" to be found in the poetry of the present age, and where does it possess a more "beautiful utterance," than in Homer's description of the interview of Hector and Andromache? Is there no sentiment in the Œdipus of Sophocles, where the blind father implores Creon, that he might be allowed to touch his children.

"Thou'lt be kind to them
For my sake, Creon; and (O latest prayer)
Let me but touch them—feel them with these hands,
And pour such sorrow as may speak farewell
O'er ills that must be theirs! By thy pure line—
For thine is pure—do this, sweet prince. Methinks
I should not miss these eyes, could I but touch them.
What shall I say to move thee?

Hast thou sent,
In mercy sent my children to my arms?
Speak—speak—I do not dream!
. . . . . . . . Blessings on thee!
For this one mercy mayst thou find above
A kinder God than I have. Ye—where are ye?
My children—come!—nearer, and nearer yet."

The *poetry* of every age is "conversant with sentiment;" and never more "principally," or elegantly so, than when Pan rendered the woods of Greece musical, and Naiads haunted the beautiful streams of that lovely land. Was not Tityrus sentimental when, under the wide-spreading beech-tree, and by the aid of his rustic pipe (tenui avená), or as Sidney Smith freely translated it, "with the aid of some thin oatmeal gruel," he made the woods to resound with the charms of the beautiful Amaryllis? Yes, that languid swain, like all his poetic brothers, was full of sentiment, and, what is more, seemed conscious of the fact.

"O Melibœe Deus nobis hec otia fecit."

The poetry of the past generation in our country—(to say no more of classic ages—the poems of Ovid, or the love-songs of Tibullus), was fully "conversant with sentiment," and found for it "a beautiful utterance" in Pope's "Abelard and Heloise," and a conspicuous and pompous display in the elaborate effusions of Edward Young. We wished to learn of some special characteristic, some defined peculiarity, which should mark a distinction between the poetry of this age and the ages which have preceded it, but we sought for it in vain in the essay before us.

We are informed that the "philosophical literature of the age is of a high order, as evidenced in the writings of Dugald Stewart and Sir John Herschel, speaking at present, as far as regards style of composition," the author reserving his profound remarks on the "*philosophy*" of the age, for another portion of his inquiry. A string of queries follows his observations upon this department of literature, as to whether "we of the present day are pigmies or giants as compared with those who have gone before us." Giants figure largely in this essay, as becomes a gigantic subject and a great author. These prodigies are brought forward in reference to the sermons of a past day, and are named three or four times in the course of a dozen lines in some parts of the work before us. It may be consolatory to the men of the nineteenth century—to the contemporaries of Chalmers and Pusey in divinity—of Adams, Le Verrier, and Herschell in astronomy—of Faraday, Davy, Berzelius, Liebig, Watt, and Wheatstone, in other departments of science, that none of those mighty questionings, big with potent thought, are answered. They are left in that solemn majesty and awful profundity which become the question and the questioner. We are rescued from any deep sense of inferiority by the fact, that Sir John Herschel himself has stated that "the intellect of Newton, La Place, or Le Grange may stand in fair competition with that of Archimedes, Aristotle, or Plato; and the virtues and patriotism of Washington with the brightest examples of ancient history."*

We have written "currente calamo," and have encroached too much upon our space before entering upon the subject which is the most deeply interesting to our readers. The author thus apostrophises the intellect, and comments upon mental philosophy.

"*The* INTELLECT! But what is intellect? And in merely asking the question, we seem suddenly sinking into a sort of abyss!"

"I asked him (an eminent metaphysician) whether he considered that we were *really* any further advanced in admitted knowledge of the nature and functions of the mind, than Aristotle was, that is, upward of twenty-two centuries ago? He considered for a moment, and replied in the negative! I then asked the same question of my other friend, and he wrote as follows: 'I am afraid very few substantial advances have been made in psychology since the days of Aristotle.' Here is a picture of existing metaphysical science! It is, in truth, only a reflection of some of the myriad dark shadows of all past speculation; and shall it be said that it bears a similar relation to the future? Metaphysics are called a science, and yet its main questions are—'What are the questions!' It deals with being and its conditions, and yet cannot say what *being* is; and indeed I doubt whether it can be truly given credit for possessing one single grand truth, universally recognised as such. . . . . A whole life of an ingenious rational being may be occupied in those pursuits—however irritating it may be to fond metaphysicians to say so—without the acknowledged acquisition of a single *fact*, of one solitary, practical, substantial result. . . . . He has been floundering on from beginnings in which nothing is begun to conclusions in which nothing is concluded," pp. 44, 45.

We have, however, omitted some important questions, and must, therefore, re-quote our author:

"But you will reasonably ask, is it then really so? A few minutes' conversation with the first professed or acknowledged metaphysician whom you meet, however he may at first dispute it, will prove the

* "Discourse on Natural Philosophy," p. 40.

existence of the fact, that the very elements of the science are floating about in extreme uncertainty. Ask him, what he means by *mind?*—is it material or immaterial? What does he understand by matter? does it exist, or not? Is thought the functional result of physical organization, or the action of a separate spiritual existence? If so, how is it united with, or what are its relations to matter? How does it stand with relation to the external world? Nay, is there any external world at all? What is the nature of the mind's internal action?" page 42.

To the important query about an "external world," we have the following "profound" foot-note:

"Bishop Berkeley, an exquisite metaphysical genius, brought profound reasonings in support of his opinion, that our belief in the reality of an external world is totally unfounded!" page 43.

We are among the "fond metaphysicians" who are "irritated" at such pretended philosophy as the above—when it is paraded as philosophy by persons possessing the social distinction and philosophic rank of the writer. We are "irritated" to think that a literary and philosophical society could patiently listen to such pedantic balderdash. Verily the "age" of gobe-mouches has not passed away. It is a vain objection to mental philosophy, that we are uncertain as to the ultimate character or quality of mind. It may *appear* philosophic to ask what *is* mind, and because it cannot be accurately defined, to sneer at all metaphysical research as empty and worthless; but is not such a proceeding the result of affectation and ignorance? Our author exhausts his great powers of eloquence, and becomes "dumb with wonder" as he contemplates the agency of steam and electricity. But, what is electricity? It has been as eloquently apostrophized, and has remained as dumb under the questionings, as has mind itself. Indeed, *all* nature, *all* science might be sneered down, if such questionings were capable of achieving such a result. If we are not to deal with any subject until we know *what* that subject *is*, in its ultimate essence, then farewell to all science; for what on earth, or sea, or sky, can we *thus* comprehend?

"This green, flowery, rock-built earth, the trees, mountains, rivers, many-sounding seas; that great deep sea of azure that swims overhead; the winds sweeping through it, the black cloud fashioning itself together, now pouring out fire, now hail and rain; what *is* it? Ay, what? At bottom, we do not yet know; we can never know at all. . . . . We call that fire of the black thunder-cloud 'electricity,' and lecture learnedly about it, and grind the like of it out of glass and silk; but *what* is it? What made it? Whence comes it? Whither goes it? Science has done much for us; but it is a poor science that would hide from us the great deep sacred infinitude of nescience, whither we can never penetrate, on which all science swims as a mere superficial film. This world, after all our science, and sciences, is still a miracle; wonderful, inscrutable, *magical* and more, to whosoever will *think* of it."*

We cheerfully admit, that man's daily necessities have acted as a stimulus to the advancement of physical science, and that the inductive method has been from an earlier date more consistently and logically applied to these studies, than to those of mental science, and that, consequently, they have arrived at greater exactitude, and to more tangible results. Mental philosophy, unfortunately, having, until within the last century, been confined mainly to colleges and schools, and even there been regarded as an "exercitation having a tendency to sharpen the faculties," rather than as earnest and grave studies, leading directly to greatly practical results;—and, therefore, no more to be charged with its present shortcomings than vaccination during the life of Jenner, or steam at the age of Hero of Alexandria, of Giovanni Branca, or the Marquis of Worcester. We would further remind, or rather inform the writer, that the speculations of Aristotle had as much relation to matter, form, and substance, as abstract speculations, as to mind; and also, that the early history of the physical sciences was fraught with speculations apparently as absurd as those directly relating to ideas—although ultimately leading to great practical results. Who could have imagined that the speculations of astrology should have merged into the science of astronomy, or that the wild dreams of the alchemists should issue in the practical facts of modern chemistry?

Mind is *now*, however, studied *inductively*, and with a view to practical results; and who beyond the range of the author's acquaintanceship is ignorant of the vast good which has sprung up from the study of psychology? Has not education been influenced by the opinions of Gall and Spurzheim—modified, corrected and improved as they have been by modern research? Has no light been thrown upon the motives to crime, and the appropriate treatment of criminals by the psychologists of the present age? Has no "practical substantial result" been "acquired by a knowledge of the deep and inseparable sympathy existing between bodily diseases and mental impulses?—Have not the insane been benefited by the mental philosophy of a Pinel, a Charlesworth, and other labourers in that great field of "practical" benevolence?—Most assuredly they have; and ignorance the most crass, or perverseness the most stolid, can alone be blind to the fact, that modern psychologists no longer hunt the shadows of an abstract speculation, more than do their contemporaries in physical science, but that both parties are

* "Carlyle, Lectures on Heroes," p. 12.

now carefully studying *facts*, and deducing from these certain "practical" conclusions, having an immediate influence upon the health and happiness of the human race. This journal is a "practical substantial result" of the advancement made in metaphysics,—and if more satisfactory evidence be demanded, we point to the efforts now being made for the improvement of the criminal code—we refer to the system of education now pursued; and triumphantly do we direct our author's attention to the absence of all manacles, fetters, and dungeons, in the various lunatic hospitals of this kingdom, and this "one solitary, practical, substantial result" is entirely due to the *inductive* study of mental phenomena. The intuitive genius of Pinel perceived in the first instance how greatly the diseases of lunatics were aggravated by a scanty supply of food, warmth, and other physical comforts, and subsequently rose to the beneficent conclusion, that chains might be dispensed with in cases where they had been worn for twenty years. With what subsequent results, let the magnificent institutions which adorn this kingdom testify. To be ignorant of the labours of Pinel and Esquirol, in France, and of Ellis and Conolly in England, as regards the influence of physical causes upon mental manifestations—to ignore the achievements of Gall and Spurzheim in educational subjects—to forget the labours of love of others who are effecting beneficent changes in medical jurisprudence, and to overlook the facts which have been accumulated in this journal by philosophic and pains-taking observers, may suit the purposes of a declamatory essay, but do not redound to the industry, penetration, or accuracy of an historian of "The Intellectual and Moral Developement of the Present Age." We do not presume to explain "how thought is united with, or what are its relations to matter," more than we pretend to unfold how *life* is so connected with organization. We rest contented with the fact, and to use the language of Goethe, "learn to know how to keep within the limits of the knowable." It may be stated, that the "substantial results" to which we have referred, did not spring from the study of "The INTELLECT;" we contend that they have, as much as the "*mechanical* power," which the author so eulogizes, has sprung up as one of "the creations of *pure intellect.*"

"No real advance in psychological science for ages," indeed! Do the facts we have related indicate no advance upon the following?

"The advocate of the fearful theory, that insanity is spiritual in its character may be represented by Luther, who said 'Idiots are men in whom devils have established themselves; and all the physicians who heal these infirmities, as though they proceeded from natural causes are ignorant blockheads, who know nothing of the power of the demon. Eight years ago, I myself saw and touched, at Dessau, a child of this sort, which had no human parents, but had proceeded from the devil,

He was twelve years old, and in outward form exactly resembled ordinary children. He did nothing but eat, consuming as much every day as four hearty labourers, or thrashers could. In most external respects, he was as I have mentioned, just like other children; but if any one touched him, he yelled out like a mad creature, and with a peculiar sort of scream. I said to the princes of Anhalt, with whom I was at the time,—if I had the ordering of things here, I would have that child thrown into the river Moldau, at the risk of being held its murderer."*

It is, however, evident from that, and other remarks, that the writer on "the Present Age" has spoken positively on a subject upon which he is less informed than the majority of his readers. We prefer the opinion of one, who has at least read the authors upon whom he presumes to speak.

"If we look back steadfastly upon the past history of philosophy, we may see that *it has ever been a progressive development;* that each age has contributed its portion, greater or less, and that the agitation between the different schools has been as it were the pulsations of the forward movement. * * * * * *

"Had Des Cartes moreover, or some equivalent mind, failed to point out the new road, Leibnitz had never trodden it, and the German philosophy were still but a possibility; and had Bacon never shown the practical power of induction, Locke had never applied it to the study of the mind, or Newton by its means furnished the key to the temple of the universe. As the course of the vessel that makes its way against the breeze consists of a series of movements, each one of which seems to bear it away from its true direction, yet brings it in fact so much further on its destined course; *so the mind that can only view each individual tack* which the philosophic spirit takes, *is apt to imagine,* that every such movement carries it *further from the true mark, whilst those who can take the whole course in at one comprehensive view, see that these apparent deviations are all necessary to bring us nearer and nearer to the centre of eternal truth.*"†

We could further *demonstrate* the "substantial result" of modern psychology in divinity, legislation, and the laws, did our space permit; but must now be content to refer the author to the pages of Brown, of Spurzheim, of Carpenter, and Holland, of Cousin, Baillarger, Esquirol, Georget, and Parchappe, if he be really desirous to know whether he was in error, when he said that "we have made no real advance in psychological science for ages," p. 51. We deeply regret that the author has joined in the vulgar habit of sneering at the "profound reasonings" of Bishop Berkeley. Every "coxcomb," according to Pope, has thought "to vanquish Berkeley with a grin;" but alas! how few have conde-

* "Hitchman on The Pathology of Insanity."—*Lancet*, 1847.
† Morell's "History of Philosophy," p. 18.

scended to read his writings. Johnson could hardly have done so when he spoke of refuting Berkeley by "kicking a stone," and we are sure, that Byron knew him not when he issued the immortal pun,

> "When Bishop Berkeley said there was no matter,
> 'Twas no matter what he said,"

inasmuch as Berkeley, *when understood,* teaches the very opposite doctrine, for to use the Bishop's *own* language—"If by matter you understand *that* which is seen, felt, tasted, and touched, then I say *matter exists:* I am as firm a believer in its existence as any one can be, *and herein I agree with the vulgar.*" Although the writer has not studied, or at least learned the "advances" which have been made in psychology, he nevertheless consoles us by the assurance "*that we have no authority from revealed religion for repressing what are called metaphysical* speculations, however little direct encouragement it may afford them," p. 50. A Genius "of the present age" has confirmed the justice, and therefore sanctioned the mercy of the olden time! Shades of Dugald Stuart, of Samuel Taylor Coleridge, and of good Bishop Berkeley, rest in peace! One of her Majesty's Counsel, a Recorder of Hull, and a Fellow of the Royal Society, has made the discovery, and proclaimed it to the world, that "there is no authority from revealed religion" to "*repress* metaphysics"—*id est,* for burning psychologists at the stake; and we are, therefore, not now called upon to supply any defect of religious zeal on the part of our forefathers by dragging you from your resting places, and burning your mouldering remains like those of a Wycliffe, as a warning, or memorial to the daring psychologists of after-generations! Happy shades! Peace be with you.

---

